# Ruptured Ectopic Pregnancy in a Non-communicating Rudimentary Horn at 18 Weeks of Gestation

**DOI:** 10.7759/cureus.76199

**Published:** 2024-12-22

**Authors:** Duaa M Al Abbas, Fatemh S Aman, Rahaf S Almuhaimeed, Zainab M Almadeh

**Affiliations:** 1 Obstetrics and Gynecology, Qatif Central Hospital, Eastern Health Cluster, Ministry of Health, Qatif, SAU; 2 Emergency, Qatif Central Hospital, Eastern Health Cluster, Ministry of Health, Qatif, SAU; 3 Medicine and Surgery, Medical University of Warsaw, Warsaw, POL; 4 Medicine, Imam Abdulrahman Bin Faisal University, Dammam, SAU; 5 Emergency, Ras Tanura General Hospital, Eastern Health Cluster, Ministry of Health, Ras Tanura, SAU

**Keywords:** ectopic pregnancy, non-communicating rudimentary horn, rudimentary horn, ruptured ectopic pregnancy, uterine anomaly

## Abstract

This case highlights the critical role of early radiological screening by ultrasound in identifying uterine anomalies. In this report, we discuss a 39-year-old pregnant woman, gravida 4 para 3, and her fetus at gestational age 18 weeks. The patient was referred to the Obstetrics and Gynecology Emergency Department at Qatif Central Hospital, Saudi Arabia, from a private hospital due to an ultrasound study indicating a possible ectopic pregnancy with an abdominal fetal location. The multidisciplinary team of obstetrics and gynecology and radiology conducted additional radiological examinations, including magnetic resonance imaging (MRI), leading to a diagnosis of suspected ruptured non-communicating rudimentary horn ectopic pregnancy. Subsequently, an emergency laparotomy was successfully performed, resulting in the removal of the rudimentary horn and the termination of the ectopic pregnancy.

## Introduction

A unicornuate uterus with a rudimentary horn is a Müllerian duct malformation that results from incomplete development of one of the Müllerian ducts and incomplete fusion with the contralateral side in embryogenesis [1]. According to the classification of uterine anomalies by the American Fertility Society, a unicornuate uterus is classified as a class II uterine anomaly and is considered to be unilateral hypoplasia or agenesis. The unicornuate uterus has four clinical presentations. It can be found as a rudimentary horn with a cavity communicating to the unicornuate uterus, a rudimentary horn with a cavity that is not communicating to the unicornuate uterus, a rudimentary horn with no cavity, or a unicornuate uterus without a rudimentary horn [2].A non-communicating rudimentary horn pregnancy is an uncommon and potentially life-threatening condition that presents significant diagnostic challenges and is often associated with complications such as uterine rupture. Early diagnosis and management are the keys to successful management and are crucial to reducing maternal mortality and morbidity [3]. In most cases, either rudimentary horn pregnancy is terminated to save the patient’s life or uterine rupture occurs in the second or third trimester, resulting in hemoperitoneum, which may be fatal. In very rare cases and under close observation, the pregnancy may continue and end with a live birth [4-6].

## Case presentation

This case report discusses a 39-year-old female patient, gravida 4, para 3, who was 18 weeks pregnant at the time of presentation. The patient had a history of regular menstrual cycles, and all previous pregnancies were spontaneous, including two uneventful full-term vaginal deliveries and, due to breech presentation, one cesarean section performed eight years prior. She was referred to our Obstetrics and Gynecology Emergency Department from a private hospital, presenting with a history of recurrent left lower groin pain since the onset of her current pregnancy. An initial ultrasound suggested the possibility of an ectopic pregnancy, indicating an abnormal fetal location within the abdomen. The patient’s general condition was stable upon examination, with vital signs within normal limits. A pelvic ultrasound was conducted, which revealed an empty uterine cavity with a clear endometrial line and no free fluid in the pouch of Douglas. Additionally, a single gestational sac containing a viable fetus with positive cardiac activity was identified in the left adnexa, further supporting the suspicion of an ectopic pregnancy.

**Figure 1:**
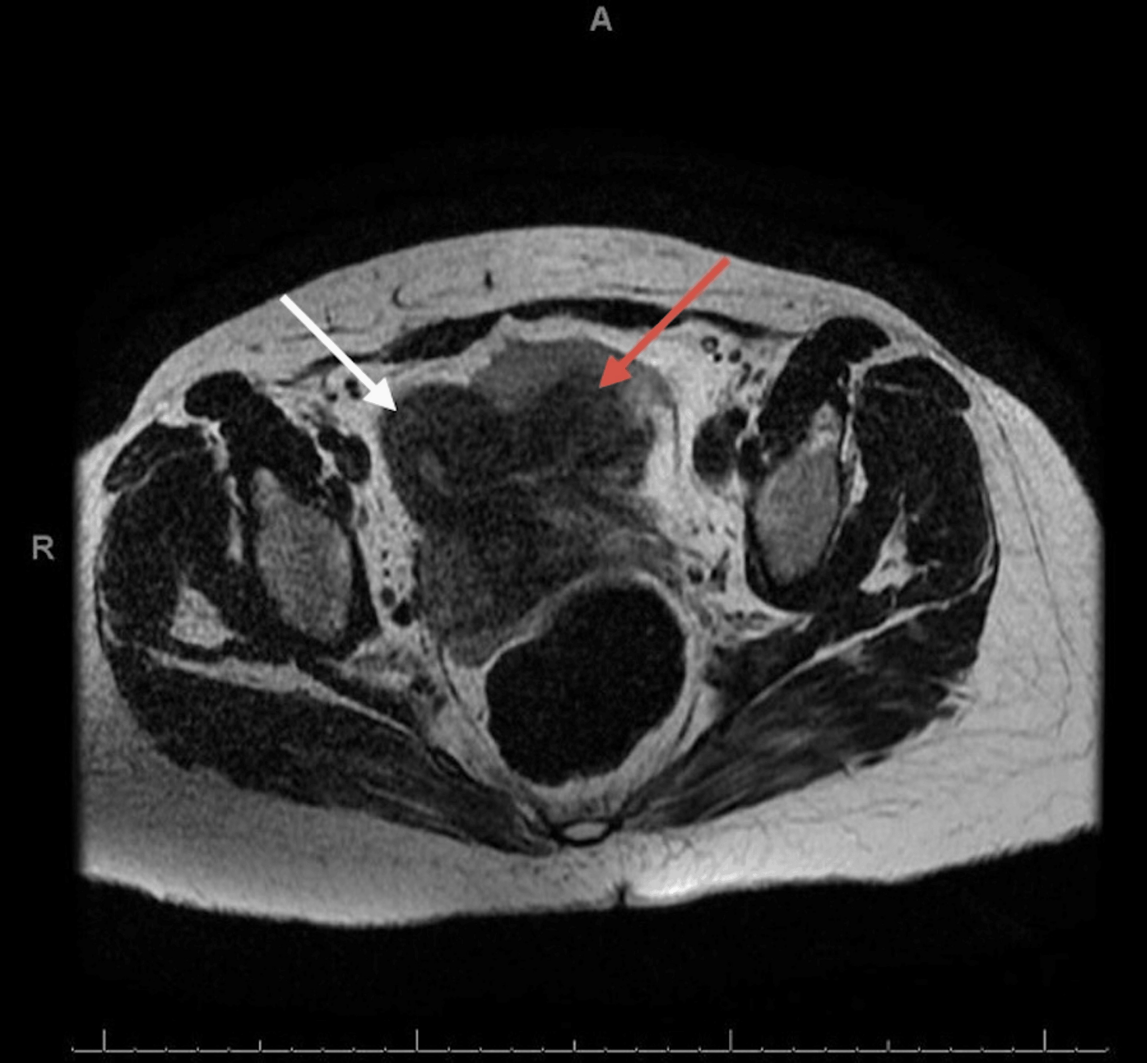
Axial image of MRI showing uterus (white arrow) with rudimentary horn (red arrow).

**Figure 2:**
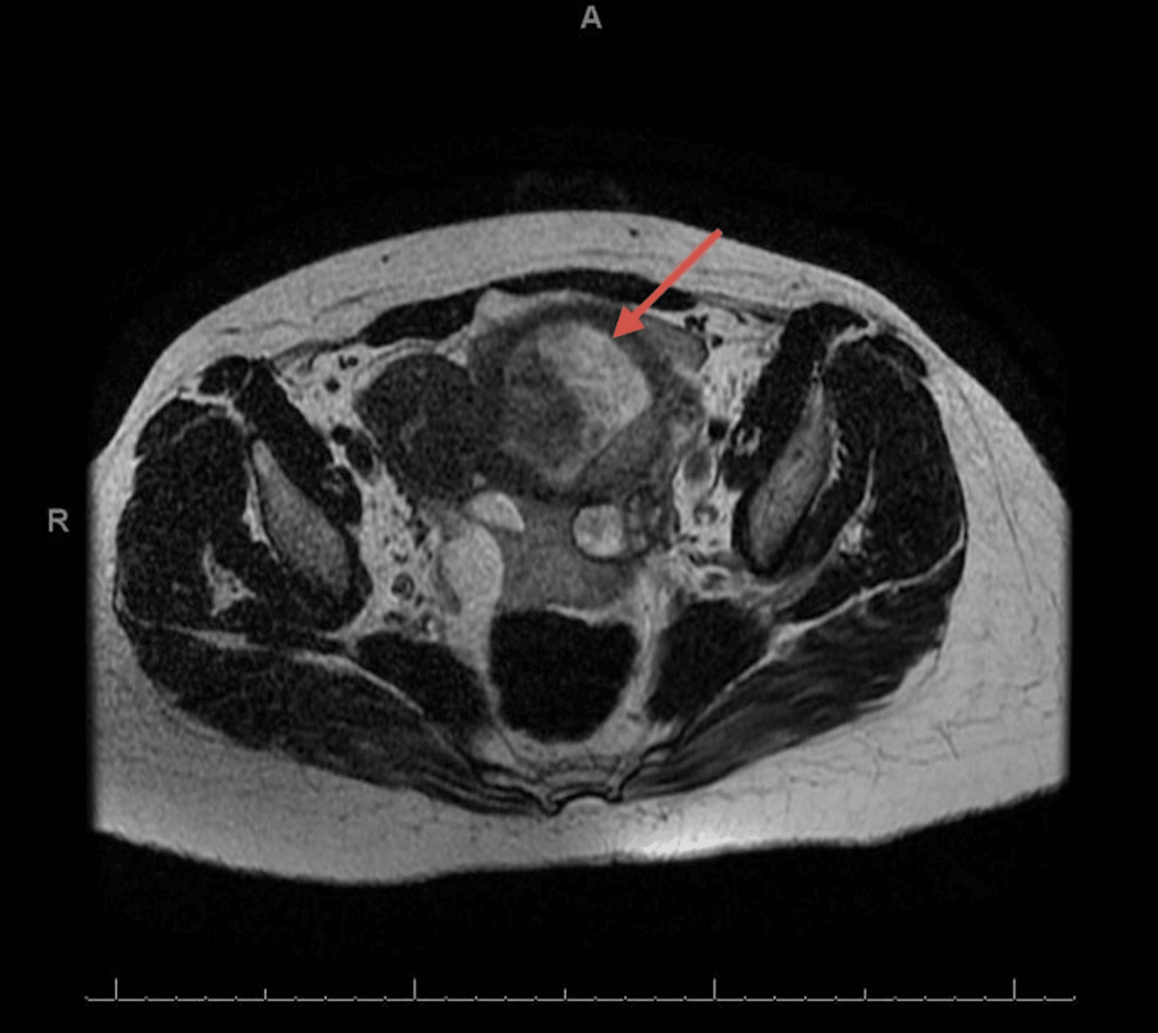
Axial image of MRI showing uterus and fetus inside the rudimentary horn (red arrow).

**Figure 3:**
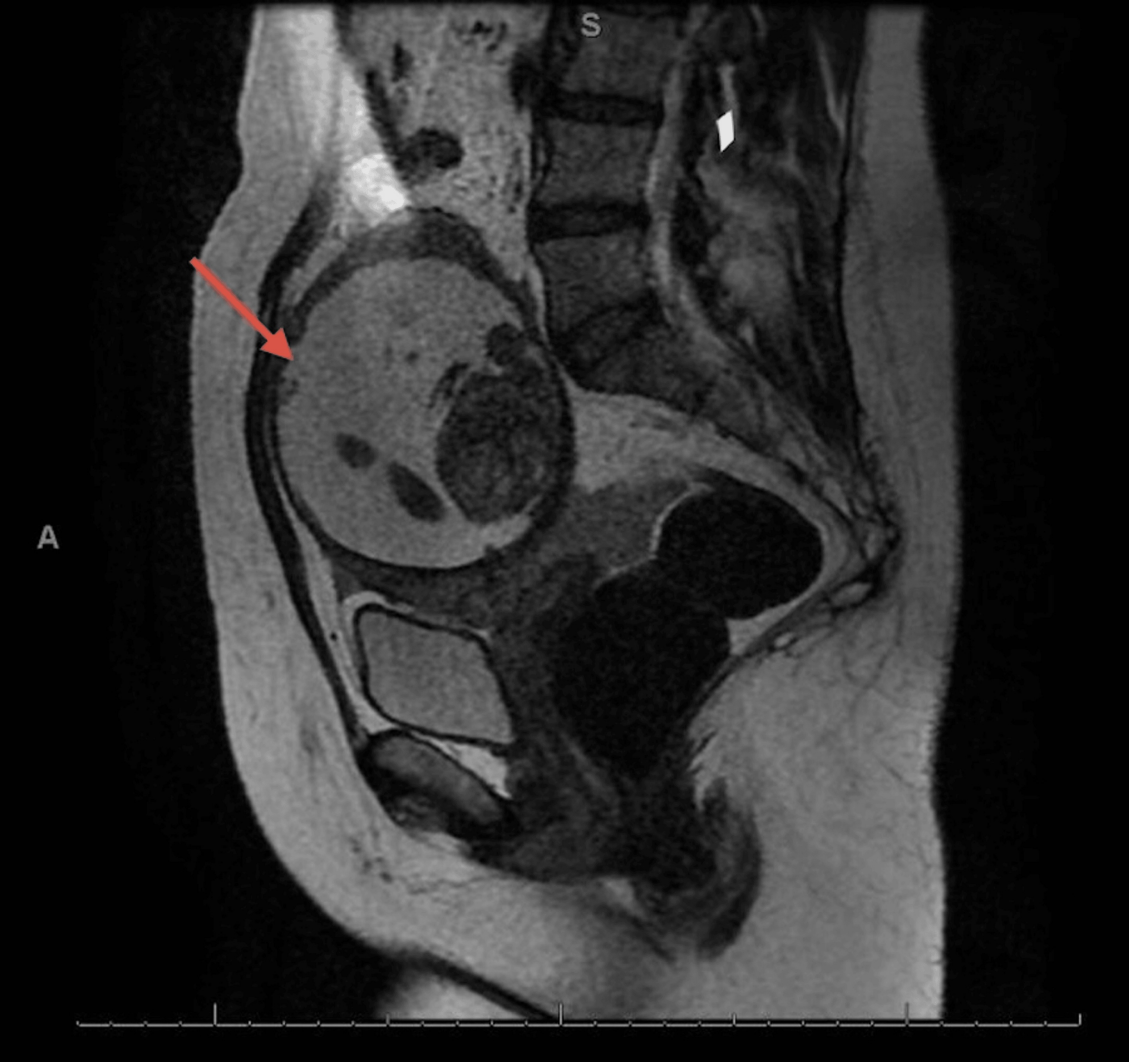
Sagittal image of MRI showing rudimentary horn pregnancy (red arrow).

Transvaginal ultrasound corroborated these findings, which confirmed an empty, normal-appearing uterus and a living fetus at 18 weeks gestation, located in the left abdominal region near the intestines, again with no free fluid present in the pouch of Douglas. Subsequently, our obstetrics and gynecology team ordered a magnetic resonance imaging (MRI) study. MRI reveals a large left pelvic ectopic pregnancy inseparable from the uterus, with surrounding low T2 signal resembling myometrial tissue and a linear hyperintense T2 signal communicating with the endometrial cavity, suggestive of a unicornuate uterus with a rudimentary horn ectopic pregnancy. The ectopic pregnancy is inseparable from adjacent small bowel loops without definitive invasion, accompanied by a small amount of complex fluid in the pouch of Douglas, suggestive of acute hemoperitoneum due to rupture. A small left dermoid ovarian cyst measuring 1.6 x 2 cm was also observed (see Figures 1-3). However, excision was not performed due to its characteristics, which closely resembled those of a follicle.

Four hours later, the patient exhibited dizziness, drowsiness, and pallor and reported experiencing sudden, intermittent pain in the left lower quadrant of the abdomen, rated as 5 out of 10 in severity. A bedside ultrasound was performed, revealing the presence of free fluid surrounding the liver and spleen along with fluid in the pouch of Douglas, thereby raising the suspicion of a potential rupture.

Surgical management

**Figure 4:**
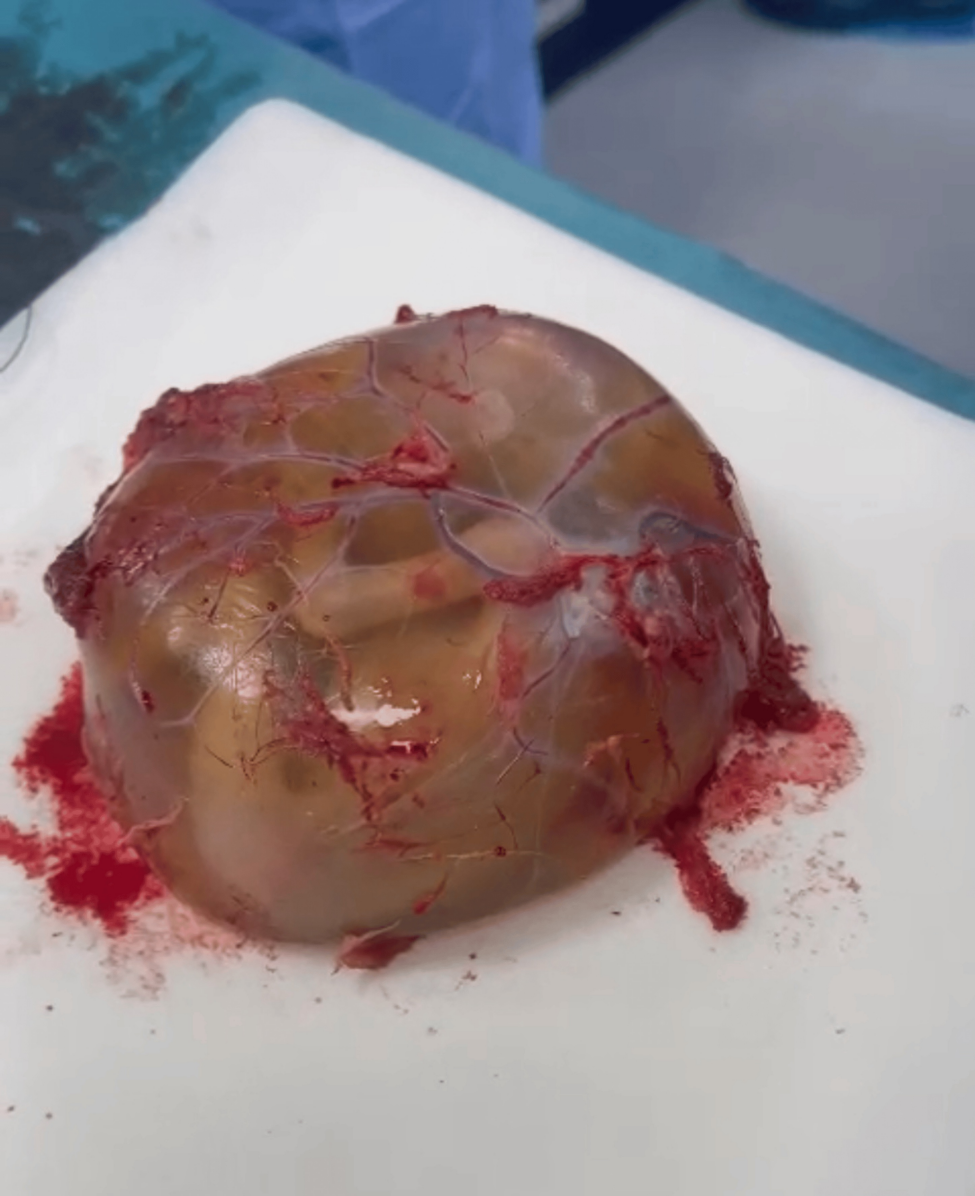
The whole amniotic sac, containing the fetus and the placenta.

**Figure 5:**
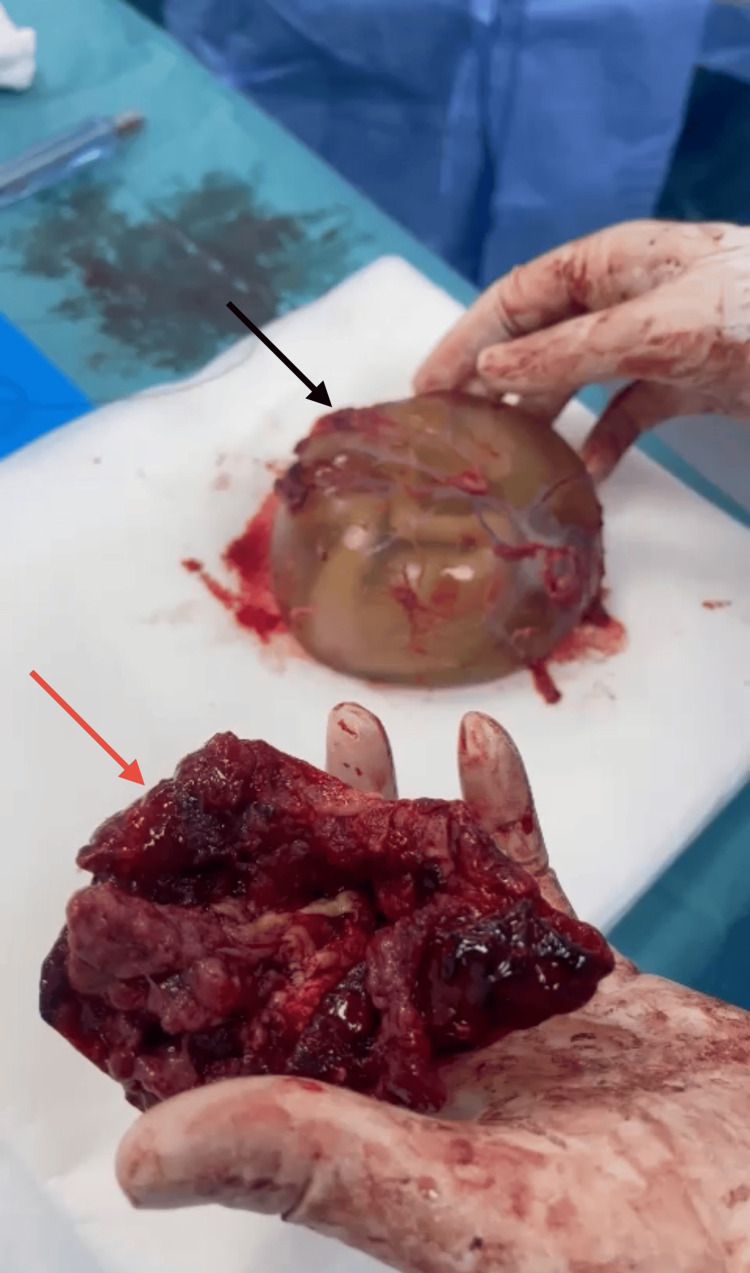
The amniotic sac with its contents (black arrow) and the resected ruptured left non-communicating rudimentary horn (red arrow).

**Figure 6:**
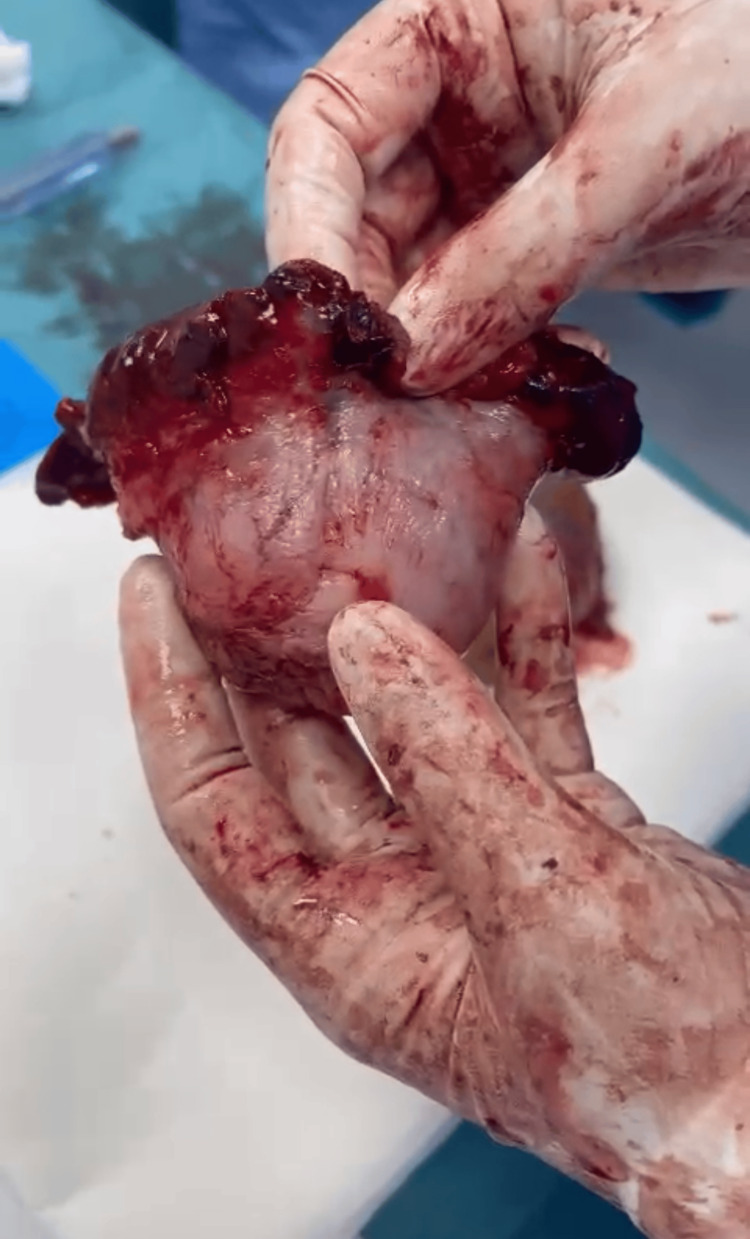
The resected ruptured left non-communicating rudimentary horn.

Under general anesthesia, the obstetrics and gynecology team conducted an emergency exploratory laparotomy. The amniotic sac, containing the fetus and placenta, was smoothly extracted without the need for dissection (see Figures 4-5). Resection of the ruptured left non-communicating rudimentary horn was successfully performed (see Figures 5-6).

The patient’s preoperative hemoglobin level was recorded at 10.1 mg/dL, while the intraoperative hemoglobin level decreased to 7.2 mg/dL. The estimated blood loss during the procedure was 100 mL. Subsequently, the patient received one unit of blood and was transferred to the recovery room in stable condition, with a postoperative hemoglobin level of 9.5 mg/dL. The patient was discharged two days after the surgery without complications, and her hemoglobin level at discharge was 10 mg/dL. Paracetamol, enoxaparin, lactulose, iron, and folic acid were prescribed as a part of the patient’s postoperative care regimen.

## Discussion

This case highlights the necessity of maintaining a high degree of clinical suspicion for such rare presentations to make the correct diagnosis and prevent complications. A transvaginal ultrasound scan (TVS) is the tool of choice for the diagnosis of ectopic pregnancies. Moreover, an MRI can help confirm the diagnosis of a rudimentary horn pregnancy. In our case, a TVS and pelvic MRI were to confirm the diagnosis. Unicornuate uterus is associated with varying degrees of chronic pelvic pain and dysmenorrhea, first-trimester miscarriage (24%), second-trimester miscarriage (9%), ectopic pregnancy (27%), preterm labor (20%), intrauterine growth retardation, intrauterine fetal death (5%), placenta accreta, and malpresentation [5]. Patients may also present with vague abdominal pain or an acute abdomen with hemorrhagic shock.

However, patients with non-communicating rudimentary horn ectopic pregnancy may present without any symptoms or with only mild symptoms, such as mild pain, as observed in our case; this can delay the diagnostic process. As a result, the patient faces a higher risk of rupture [7]. In some cases, a rudimentary horn can be misdiagnosed as other types of uterine masses, such as a subserosal uterine fibroid, as observed in our case, which makes the diagnosis more challenging [8,9].

In this case, the uterine horn was misdiagnosed as a subserosal uterine fibroid during the patient’s previous cesarean section delivery. If the uterine horn had been discovered and diagnosed correctly during the previous cesarean section or the previous pregnancy, surgical removal of the horn would have been initiated, preventing the ectopic rudimentary uterine horn pregnancy. The outcomes of a uterine horn pregnancy can include abortion, preterm delivery, uterine rupture, and, rarely, full-term pregnancy. Early detection of rudimentary horn pregnancies is crucial for preventing and managing life-threatening complications, as these pregnancies are associated with high maternal mortality and morbidity. The risk of uterine rupture can reach up to 50%, which may lead to hemoperitoneum and potentially result in death [4]. The treatment of choice for a rudimentary horn pregnancy is surgical excision of the rudimentary horn. Laparotomy is performed to prevent the risk of possible catastrophic consequences due to rupture. The use of preoperative methotrexate to terminate early pregnancy before laparoscopic intervention has also been reported in the literature [10,11].

In the case of our patient, we resected the rudimentary horn via laparotomy. It is recommended not to delay surgery once the diagnosis of an unruptured ectopic pregnancy in a rudimentary horn is made, as the timing of rupture depends on the thickness of the horn musculature [12]. Our patient made an uneventful recovery, and the doctor discharged her home in good condition. The patient attended her follow-up appointment one month later, exhibiting favorable progress. The theory of sperm transmigration from the contralateral oviduct has been advocated by Ansari and Miller [13], with confirmation provided by the presence of a corpus luteum in the ipsilateral adnexa. Our finding that no “tunnel” connects the rudimentary horn with the unicornuate cavity supports this proposition. Close follow-up of subsequent pregnancies in such cases is recommended due to the retention of the rudimentary horn and fallopian tube [13,14]. There have been reports of successful pregnancy and term delivery following the treatment of a unicornuate uterus with a non-communicating rudimentary horn pregnancy, and these treatments involved local methotrexate injection followed by laparoscopic resection [13,14].

## Conclusions

Non-communicating rudimentary horn pregnancy presents significant diagnostic challenges due to its atypical presentation and the potential for severe complications, including uterine rupture. Early and accurate diagnosis, facilitated by comprehensive imaging techniques, is crucial in managing these cases. It is essential to prompt surgical intervention to prevent life-threatening outcomes and ensure patient safety. This case highlights the importance of clinical awareness and vigilance in the early identification and treatment of rare uterine anomalies.

Following the surgical removal of a rudimentary uterine horn, patients require comprehensive counseling and education that addresses their medical and emotional needs. It is crucial to discuss the risks of future pregnancies, including uterine rupture, and to recommend temporary contraception until the patient receives medical clearance to conceive. Close monitoring is essential for any subsequent pregnancies.
